# Impact of Sodium-Glucose Cotransporter-2 Inhibitors on Heart Failure in Patients With Type 2 Diabetes Mellitus: A Systematic Review

**DOI:** 10.7759/cureus.68560

**Published:** 2024-09-03

**Authors:** Hala A Abdelhady, Adoum Oumar Abakar, Ravindra Reddy Gangavarapu, Sayed A Mahmud, Anura Manandhar, Ghadeer Sabir, Iana Malasevskaia

**Affiliations:** 1 Internal Medicine, California Institute of Behavioral Neurosciences & Psychology, Fairfield, USA; 2 Internal Medicine, Universidad de Ciencias Medicas de La Habana, Havana, CUB; 3 Medical Research, California Institute of Behavioral Neurosciences & Psychology, Fairfield, USA; 4 Research and Development, California Institute of Behavioral Neurosciences & Psychology, Fairfield, USA; 5 Obstetrics and Gynecology, Private Clinic 'Yana Alexandr', Sana'a, YEM

**Keywords:** t2dm, diabetes mellitus type 2, left ventricular dysfunction, congestive heart failure, heart failure, canagliflozin, empagliflozin, dapagliflozin, sglt2 inhibitors, sodium-glucose cotransporter-2 inhibitors

## Abstract

Type 2 diabetes mellitus (T2DM) is a major global health concern with a strong association with increased cardiovascular morbidity and mortality. The prevalence of heart failure is significantly higher in the T2DM population compared to non-diabetic individuals. Sodium-glucose cotransporter-2 (SGLT-2) inhibitors have emerged as a promising therapeutic class for managing T2DM, with potential cardioprotective effects. This systematic review aims to comprehensively evaluate the impact of SGLT-2 inhibitors on cardiovascular outcomes in adult patients with T2DM. A comprehensive electronic search was conducted across multiple databases and registries from May 8 to June 6, 2024, following the Preferred Reporting Items for Systematic Reviews and Meta-Analyses (PRISMA) 2020 guidelines. Studies published between January 2019 and June 6, 2024 that evaluated the effects of SGLT-2 inhibitors on cardiovascular outcomes in adults with T2DM were included. The risk of bias was assessed using appropriate tools based on the study design. A narrative synthesis was planned to summarize the findings. The search strategy identified 25 studies (22 randomized controlled trials, three cohort studies) for inclusion in the systematic review. Most of the included studies demonstrated a low overall risk of bias, although some observational studies had some limitations. The studies investigated the effects of various SGLT-2 inhibitors, including empagliflozin, canagliflozin, dapagliflozin, and others, on cardiovascular endpoints such as heart failure-related hospitalizations, mortality, cardiac structure and function, and biomarkers. The findings suggest that SGLT-2 inhibitors may have a beneficial impact on reducing the risk of heart failure-related hospitalizations and potentially improving other cardiovascular outcomes in patients with T2DM. This comprehensive systematic review provides valuable insights into the emerging role of SGLT-2 inhibitors in mitigating cardiovascular complications associated with T2DM. The findings have important clinical implications and may inform evidence-based guidelines and treatment strategies aimed at improving cardiovascular outcomes in this high-risk patient population.

## Introduction and background

Type 2 diabetes mellitus (T2DM) is a major worldwide health problem that directly impacts the integrity of blood vessels of different sizes all over the body, resulting in macro- and microangiopathies [1-2]. In heart failure cohort studies, the prevalence of T2DM was found to range between 10% and 47%, with rates exceeding 40% in patients requiring hospitalization for decompensated heart failure, while in studies of T2DM, heart failure prevalence ranged between 9% and 22%, which is believed to be four-folds higher than in the general population. Some observational studies reached the same conclusion as well [3]. Left ventricular dysfunction (LVD), a marker of compromised heart function, is a frequent complication in patients with T2DM, even in the absence of established cardiovascular diseases like hypertension or coronary insufficiency [2]. While various cardioprotective therapies exist, the risk of developing heart failure remains significantly higher in the T2DM population compared to non-diabetic individuals [4]. Sodium-glucose cotransporter-2 inhibitors (SGLT-2 inhibitors) represent a novel class of medications used for T2DM management due to their blood sugar-lowering effects. Beyond their primary hypoglycemic action, SGLT-2 inhibitors have been shown to positively impact cardiovascular health by potentially reducing blood pressure, intravascular volume, and body weight, all factors considered cardioprotective [2]. Recent research has explored the potential of SGLT-2 inhibitors in mitigating cardiovascular complications associated with T2DM, including heart failure progression, hospitalization rates, and mortality [4]. This systematic review aims to comprehensively evaluate the impact of SGLT-2 inhibitors on cardiovascular outcomes in adult patients with T2DM within the past five years.

## Review

This systematic review was conducted following the Preferred Reporting Items for Systematic Reviews and Meta-Analyses (PRISMA) 2020 guidelines [5].

Search strategy

A comprehensive electronic search was conducted across multiple databases and registers between May 8 and June 6, 2024. These included PubMed/MEDLINE, Cochrane Library, Google Scholar, ScienceDirect, International Standard Registered Clinical Trial Number (ISRCTN) ClinicalTrial.gov registries, and ResearchGate.

The search strategy combined Medical Subject Headings (MeSH) terms and Boolean operators (AND, OR, NOT) to identify relevant studies. Three key concepts were used for the search: "SGLT-2 inhibitors," "Heart failure," and "Diabetes mellitus, type 2." The detailed search strategy for each database is presented in Table 1.

**Table 1:** Search Strategy Used to Identify Studies on SGLT-2 Inhibitors and Heart Failure in Type 2 Diabetes SGLT-2 inhibitors: sodium-glucose cotransporter 2 inhibitors; ISRCTN: International Standard Randomized Controlled Trial Number; Mesh: Medical Subject Headings.

Search strategy	Database used
"Sodium-Glucose Transporter 2 Inhibitors/therapeutic use"[Mesh] OR "SGLT2 Inhibitors" OR "SGLT 2 Inhibitors" OR "SGLT 2 Inhibitor" OR "SGLT-2 Inhibitor" OR "SGLT-2 Inhibitors" OR "Sodium Glucose Transporter 2 Inhibitors" OR "Sodium-Glucose Transporter 2 Inhibitor" OR "Gliflozin" OR "SGLT2 Inhibitor" OR "Gliflozins" OR "Sodium Glucose Transporter 2 Inhibitor" AND "Heart Failure/drug therapy"[Mesh] OR "Heart Failure/prevention and control"[Mesh] OR "Heart Failure/therapy"[Mesh] OR "Cardiac Failure" OR "Heart Decompensation" OR "Heart Failure" OR "Myocardial Failure" OR "Congestive Heart Failure" OR "Left Sided Heart Failure" OR "Right Sided Heart Failure" AND "Diabetes Mellitus/drug therapy"[Mesh] OR "Diabetes Mellitus/therapy"[Mesh] OR "Diabetes Mellitus" OR "Type 2 Diabetes Mellitus" OR "Diabetes Mellitus, Adult-Onset" OR "Non-Insulin-Dependent; Diabetes Mellitus" OR "Diabetes Mellitus, Type II" OR "Ketosis-Resistant Diabetes Mellitus"	PubMed advanced search + Medline
[Sodium-Glucose Transporter 2 Inhibitors] explode all trees OR ("sodium glucose co-transporter 2 inhibitor"):ti,ab,kw AND Heart Failure] explode all trees OR ("heart-failure"):ti,ab,kw AND [Diabetes Mellitus, Type 2] explode all trees OR ("diabetes mellitus type 2"):ti,ab,kw	Cochrane Library
allintitle: SGLT2 inhibitors AND heart failure AND diabetes mellitus type 2 -"review" -"meta analysis"	Google Scholar
SGLT2 inhibitors AND "heart failure" AND "diabetes Mellitus type 2"	Science Direct
Dapagliflozin AND heart failure	ISRCTN registry
sodium glucose cotransporter 2 inhibitors AND Heart failure	ResearchGate
sodium-glucose cotransporter 2 inhibitors AND Heart failure	ClinicalTrials.Gov registry

Study selection

Our study selection process was transparent and meticulously detailed. Inclusion and exclusion criteria, outlined in Table 2, were established before the eligibility of retrieved studies was ensured. Titles and abstracts of identified studies were screened by a single reviewer (H.A.) based on the pre-defined criteria. Studies deemed potentially relevant underwent a full-text review by two independent reviewers (H.A. and A.M). Disagreements were resolved through discussion or consulting a third reviewer (I.M.), ensuring the integrity and transparency of our research.

**Table 2:** Inclusion and exclusion criteria HFrEF: heart failure with reduced ejection fraction; HFpEF: heart failure with preserved ejection fraction; DM type 2: diabetes mellitus type 2; RCTs: randomized control trials; CCTs: controlled clinical trials.

	Inclusion criteria	Exclusion criteria
Date	Studies conducted between January 2019 and June 6, 2024	Before 2019
Population	Adults (>18 years) diagnosed with DM type 2 and heart failure (HFrEF or HFpEF) treated with sodium-glucose co-transporter II inhibitor agents	Studies on animal, non-diabetic patients.
Publication type	Original studies (peer-reviewed) and Registers	
Study design	RCTs, CCTs, Observational studies	Reviews, meta-analyses, case reports, editorials, commentaries, abstracts, uncompleted studies, completed studies without results
Location	International literature	
Language	English	Not in English

Data extraction and quality assessment

Data extraction was conducted by H.A. using a standardized data collection form. Extracted data included study characteristics, participant demographics, interventions, outcomes, and risk of bias assessments. The quality of the included studies was independently assessed by two reviewers (H.A. and A.M.) using appropriate tools. The specific tools used depend on the study design, Cochrane Risk of Bias tool 2 (ROB 2) for RCTs [6], Joanna Briggs Institute (JBI) critical appraisal checklist for non-randomized studies [7], and Newcastle-Ottawa Scale (NOS) for cohort studies [8]. Any discrepancies were resolved through discussion or consulting a third reviewer (I.M.).

Data management and synthesis

Extracted data was managed using reference management software (Zotero: Corporation for Digital Scholarship at George Mason university, Fairfax Virginia, USA). A narrative synthesis was conducted to summarize the findings of the included studies.

Results

Databases and Registers Search Results

A comprehensive search strategy identified 376 studies across PubMed/Medline, Cochrane Library, Google Scholar, and ScienceDirect, along with 100 studies from ClinicalTrials.gov and ISRCTN and 20 studies identified through the ResearchGate website. Duplicates were removed using Zotero, resulting in a reduced pool of 232 studies. These studies were then screened by title and abstract, resulting in 74 studies selected for full-text review.

**Figure 1:**
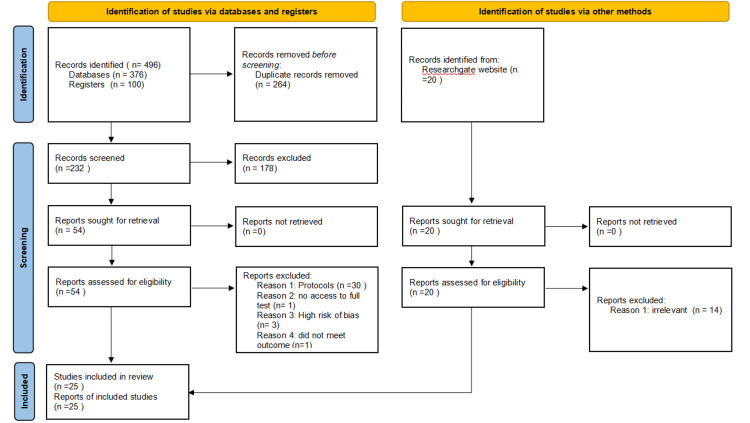
PRISMA Flow Diagram PRISMA: Preferred Reporting Items for Systematic Reviews and Meta-Analyses.

Following full-text assessment, 45 studies were excluded. These exclusions comprised 30 protocols, three studies deemed to have high risk of bias, one that was inaccessible for full evaluation, and one that did not meet the predetermined inclusion criteria. A total of 25 studies were ultimately included in the review (detailed in the PRISMA flow diagram, Figure 1).

Risk of Bias Assessment Results

The included studies' methodological quality was evaluated using appropriate tools based on their design. The RCT studies (n=22) were evaluated using the Cochrane Risk of Bias Tool 2 (Table 3).

**Table 3:** Results of the Cochrane Risk of Bias 2 Assessment for Included Randomized Clinical Trials D1: bias arising from the randomization process, D2: bias due to deviations from intended interventions, D3: Bias due to missing outcome data, D4: bias in the measurement of the outcome, D5: bias in the selection of the reported result.

Authors and year	D1	D2	D3	D4	D5	The overall risk of bias
Tanaka et al., 2020 [9]	Low risk	Some concerns	Low risk	Low risk	Low risk	Some concerns
Kayano et al., 2020 [10]	Some concerns	Low risk	Low risk	Low risk	Low risk	Some concerns
Palau et al., 2022 [11]	Low risk	Low risk	Low risk	Low risk	Low risk	Low risk
Inzucchi et al., 2020 [12]	Low risk	Low risk	Low risk	Low risk	Low risk	Low risk
Yeoh et al., 2020 [13]	Low risk	Low risk	Low risk	Low risk	Low risk	Low risk
Singh et al., 2020 [14]	Low risk	Some concerns	Low risk	Low risk	Low risk	Some concerns
Pratley et al., 2023 [15]	Low risk	Low risk	Low risk	Low risk	Low risk	Low risk
Griffin et al., 2020 [16]	Low risk	Low risk	Low risk	Low risk	Some concerns	Some concerns
Butt et al., 2022 [17]	Low risk	Low risk	Low risk	Low risk	Low risk	Low risk
Pitt et al., 2023 [18]	Low risk	Low risk	Low risk	Low risk	Low risk	Low risk
Berg et al., 2019 [19]	Low risk	Low risk	Low risk	Low risk	Low risk	Low risk
Kato et al., 2019 [20]	Low risk	Low risk	Low risk	Low risk	Low risk	Low risk
Solomon et al., 2022 [21]	Low risk	Low risk	Low risk	Low risk	Low risk	Low risk
Furtado et al., 2019 [22]	Low risk	Low risk	Low risk	Low risk	Low risk	Low risk
Wiviott et al., 2019 [23]	Low risk	Low risk	Low risk	Low risk	Low risk	Low risk
Anker et al., 2021 [24]	Low risk	Low risk	Low risk	Low risk	Low risk	Low risk
Lee et al., 2021 [25]	Low risk	Low risk	Low risk	Low risk	Low risk	Low risk
Krämer et al., 2023 [26]	Low risk	Low lack	Low risk	Low risk	Some concerns	Some concerns
Sen et al., 2021 [27]	Low risk	Low risk	Low risk	Low risk	Low risk	Low risk
Ferreira et al., 2021 [28]	Low risk	Low risk	Low risk	Low risk	Low risk	Low risk
Bhatt et al., 2021 [29]	Low risk	Low risk	Some concerns	Some concerns	Some concerns	Some concerns
Nassif et al., 2019 [30]	Low	Low	Low	Low	Low	Low

While most RCTs demonstrated a low risk of bias, four studies warranted closer examination. Tanaka et al. (2020) [9] and Singh et al. (2020) [14], both open-label studies, raised concerns in a specific domain (Domain 2) due to the lack of blinding (Table 3). This absence of blinding could potentially introduce performance and detection bias. Similarly, Griffiń et al. (2020) [16] and Krämer et al. (2023) [26] raised concerns in Domain 5 (bias in selecting reported results) by conducting multiple post hoc subgroup analyses without adjusting for multiple comparisons. This approach could inflate the risk of selective reporting bias. Furthermore, the study by Kayano et al. (2020) [10] raised concerns about potential issues in Domain 1 (randomization process) that could elevate the overall risk of bias in their study. Moreover, Bhatt et al. 2021 [29] raised concerns about potential bias due to difficulty blinding participants and incomplete outcome data in the SOLOIST-WHF trial.

Table 4 presents the quality assessment of included observational cohort studies (n=3) using the Newcastle-Ottawa Scale (NOS). The NOS is a tool designed to evaluate the methodological rigor of non-randomized studies. It focuses on three key areas that can introduce bias: selection, comparability, and outcome. Selection (maximum four stars) assesses how well the study minimizes bias in choosing participants. Comparability (maximum two stars) evaluates how effectively the study accounts for differences between exposed and unexposed groups at the outset. Finally, the outcome (maximum three stars) examines how well the study measures the outcome of interest and minimizes bias in its ascertainment. Each study receives stars based on specific criteria within each domain. A higher total score (maximum nine stars) indicates a more robust overall study design.

**Table 4:** Results of the Newcastle-Ottawa Scale for Included Observational Cohort Studies

Authors and year	Selection (max 4)	Comparability (max 2)	Outcome (max 3)	Total Score (max 9)
Norhammar et al., 2019 [4]	⭐⭐⭐⭐	⭐⭐	⭐⭐⭐	⭐⭐⭐⭐⭐⭐⭐⭐⭐
Real et al., 2021 [31]	⭐⭐⭐⭐	⭐⭐	⭐⭐⭐	⭐⭐⭐⭐⭐⭐⭐⭐⭐
Jariwala et al., 2021 [32]	⭐⭐⭐	-	⭐⭐⭐	⭐⭐⭐⭐⭐⭐

While Table 4 shows two studies with high methodological quality (nine stars), the remaining studies fall into the moderate quality range (six stars) based on the Newcastle-Ottawa Scale assessment. The studies by Norhammar et al. (2019) [4] and Real et al. (2021) [31] achieved the highest scores (nine stars), indicating robust methodology. Conversely, the study by Jariwala et al. (2021) [32] (six stars) exhibited lower scores, suggesting potential limitations received lower scores in outcome assessment, possibly indicating limitations in measuring or controlling for confounding factors (Table 4).

The ROBINS-I tool was implemented to assess the quality of non-randomized trials (n=3), revealing varying quality (Table 5).

**Table 5:** Results of the Risk of Bias in Non-randomized Studies of Interventions (ROBINS I) of Included Non-randomized Studies D1: bias due to confounding, D2: bias in the selection of participants in the study, D3: bias in the classification of interventions, D4: bias due to deviations from intended interventions, D5: bias due to missing data, D6: bias in the measurement of outcomes, D7: bias in the selection of the reported result.

Author and year	D1	D2	D3	D4	D5	D6	D7	Overall risk of bias
Sezai et al., 2019 [33]	Serious risk of bias	Low	Low	Low	Low	Moderate	Low	Serious risk of bias
Nakagaito et al., 2019 [34]	Serious risk of bias	Low	Low	Moderate	Low	Low	Low	Serious risk of bias
Ozisik et al., 2021 [35]	Serious risk of bias	Low	Low	Moderate	Low	Moderate	Low	Serious risk of bias

All non-randomized clinical trials had an overall severe risk of bias due to the lack of a control group and inadequate adjustment for confounding factors (Table 5). To ensure the strength and high quality of our analysis moving forward, we excluded studies with a high risk of bias. This exclusion allows us to focus on the most reliable evidence available. Consequently, most of the included studies, particularly the well-designed RCTs, had a low risk of bias and can be considered high-quality evidence. However, some observational studies had more limitations and potential sources of bias that should be considered when interpreting their findings.

Study Characteristics

Our comprehensive review analyzed 25 studies investigating the impact of SGLT-2 inhibitors on cardiovascular outcomes in a total number of 157,998 patients with T2DM. These studies included 22 RCTs and three cohort studies. The patient populations varied in terms of baseline characteristics, with some studies focusing on individuals with established heart failure, while others included a broader spectrum of T2DM patients. The specific SGLT-2 inhibitors used also differed across the studies. Despite this heterogeneity, the studies collectively provide valuable insights into the potential cardiovascular benefits of SGLT-2 inhibitors in this patient population. Table 6 presents a detailed breakdown of the included studies, highlighting their key characteristics, methodologies, primary outcomes, and secondary outcomes.

**Table 6:** Characteristics of Included Studies 3P-MACE: 3-point Major Adverse Cardiovascular Events (includes heart attack, stroke, and cardiovascular death); NT-proBNP: N-terminal pro-brain natriuretic peptide; MI: myocardial infarction; GDF‐15: growth differentiation factor‐15; LVESVi: left ventricular end-systolic volume indexed; LV GLS: left ventricular global longitudinal strain; LVEDVi: left ventricular end-diastolic volume indexed; GLDs: glucose-lowering drugs; oGLDs: other glucose-lowering drugs; ARNI: angiotensin receptor blocker-neprilysin inhibitors; CANDLE trial: Cardiovascular and Mortality Benefits of Canagliflozin in Patients with Type 2 Diabetes and Chronic Heart Failure; DAPA-VO2 trial: Short-term Effects of Dapagliflozin on Peak VO2 in HFrEF trial; EMPA-REG OUTCOME Trial: (Empagliflozin) Cardiovascular Outcome Event Trial in Type 2 Diabetes Mellitus Patients trial; DAPA-HF trial: Dapagliflozin And Prevention of Adverse outcomes in Heart Failure trial; REFORM Trial: Dapagliflozin Versus Placebo on Left Ventricular Remodeling in Patients With Diabetes and Heart Failure trial; SOLOIST-WHF trial: Sotagliflozin in Patients with Diabetes and Recent Worsening Heart Failure trial; VERTIS trial: Cardiovascular Outcomes Following Ertugliflozin Treatment in Type 2 Diabetes Mellitus Participants With Vascular Disease trial; DECLARE-TIMI 58 trial: Dapagliflozin Effect on Cardiovascular Events; EMPEROR-Reduced trial: Empagliflozin Outcome Trial in Patients With Chronic Heart Failure With Reduced Ejection Fraction; SUGAR-DM-HF trial: Empagliflozin and its Cardiovascular, Renal and Metabolic Effects in Patients With Diabetes Mellitus, or Prediabetes, and Heart Failure trial; DEFINE-HF trial: Dapagliflozin Effects on Biomarkers, Symptoms, and Functional Status in Patients With Heart Failure With Reduced Ejection Fraction trial; CVD-REAL Catalonia study: Cardiovascular Disease-REal-world Analysis study; RCT: randomized control trial; DBP: diastolic blood pressure.

Authors and year	Aim of study	Study type	N of participants	Intervention/exposure treatment duration	Results/Conclusion
Tanaka et al., 2020 [9]	Evaluation of the effect of Canagliflozin compared to glimepiride on the HF biomarker (NT-proBNP) in T2DM patients	RCT (CANDLE trial)	233	Canagliflozin 100 mg/day vs Glimepiride (starting dose: 0.5 mg/day) increase of up to 6.0 mg/day 24 weeks	Primary endpoint (non-inferiority for NT-proBNP): p-value = 0.226; NT-proBNP in canagliflozin group: p-value = 0.087; NT-proBNP in the subgroup with preserved left ventricular ejection fraction (LVEF) left ventricular end-systolic volume: p-value = 0.098. Canagliflozin was not definitively non-inferior to glimepiride for NT-proBNP changes.
Kayano et al., 2020 [10]	Evaluation of sodium-glucose cotransporter-2 inhibitors (SGLT2-i) effects on left ventricular (LV) pump function, LV filling pressure (LVFP), and right ventricular systolic pressure (RVSP) during exercise in T2DM patients	RCT	78	Dapagliflozin (D) 5mg/day (add-on) vs Conventional therapy (C) (add-on) 6 months	A significant decrease in left ventricular filling pressure (LVFP), blood pressure, and right ventricular systolic pressure (RVSP) between baseline and 6-month follow-up was noticed in the D-group (p<0.001) compared to Group C. No significant change in stroke volume index (SVi; mL/m2), and cardiac index in both groups. Dapagliflozin improved exercise hemodynamics in T2DM patients with cardiovascular (CV) risk, suggesting a potential benefit for HF management.
Palau et al., 2022 [11]	Evaluation of the effect of dapagliflozin on 1- and 3-month maximal functional capacity in patients with stable HF with reduced ejection fraction (HFrEF).	(DAPA-VO2) trial RCT	90	Dapagliflozin 10 mg/day vs placebo 3 months	Peak oxygen consumption (VO_2_) significantly increased in patients receiving dapagliflozin at both 1 month (p=0.021) and 3 months (p=0.032). No significant changes were observed in other measured outcomes (6-minute walk test, quality of life score, echocardiographic parameters). Dapagliflozin treatment in patients with stable HFrEF significantly increased peak oxygen consumption (VO_2_) suggesting improved exercise capacity.
Inzucchi et al., 2020 [12]	Assess if SGLT2 inhibitor empagliflozin benefits CV health in diabetics, and if managing other risk factors enhances this effect.	Post hoc analysis of RCT EMPA-REG OUTCOME Trial	7,020	Empagliflozin 10 mg, empagliflozin 25 mg, or placebo once daily. Continue until at least 691 patients have experienced an event: 3-point major adverse cardiovascular events (3P-MACE).	Empagliflozin significantly reduced CV risk death, HF hospitalization, major adverse CV events) in patients with T2DM, regardless of how well-controlled their other CV risk factors were at baseline (p>0.05 for all interactions). Empagliflozin offers significant CV benefits for diabetics, even if other risk factors are not perfectly controlled.
Yeoh et al., 2020 [13]	To compare baseline patient characteristics, outcomes, and the efficacy and safety of dapagliflozin, in relation to time from diagnosis of heart failure (HF) in DAPA-HF trial	Post hoc analysis of DAPA-HF, of RCT	4,744	Dapagliflozin 10 mg once daily, added to standard care vs matching placebo.	Sicker patients with longer heart failure duration still benefited from dapagliflozin treatment (HR=0.64 for >5 years). The benefit was consistent across HF duration (p-interaction=0.26). Sicker patients with longer-lasting HF still benefited from dapagliflozin treatment.
Singh et al., 2020 [14]	Effect of Dapagliflozin on left ventricular remodeling assessed by cardiac MRI in diabetic patients with HF	RCT (The REFORM Trial)	56	Dapagliflozin 10 mg daily vs placebo 1 year	Dapagliflozin did not improve left ventricle remodeling (left ventricular End-systolic volume (LVESV) unchanged), but offered other benefits: Lowered DBP, reduced need for loop diuretics (potentially due to fluid loss). Increased hemoglobin and hematocrit, increased ketone bodies, possible weight loss trend. Dapagliflozin's impact on LV remodeling in T2DM with HF remained unclear.
Pratley et al., 2023 [15]	Effect of Ertugliflozin on cardiorenal outcomes across different age groups	The secondary analyses of (VERTIS CV) RCT	8,246	Ertugliflozin 5 mg once a day vs ertugliflozin 15 mg once a day vs or placebo	No increased risk of heart attacks, heart death, or kidney problems compared to placebo (p>0.05). Ertugliflozin reduced hospitalizations for HF. Slower decline in kidney function with ertugliflozin compared to placebo. Ertugliflozin seems safe and potentially beneficial for heart and kidney health in older adults with T2DM and heart disease.
Griffin et al., 2020 [16]	To investigate the diuretic properties of empagliflozin in patients with HF and T2DM	Crossover RCT	20	Empagliflozin 10 mg daily vs placebo 14 days with a two-week washout period in between for a total of 28 days per participant	Empagliflozin significantly increased natriuresis compared to placebo (p<0.0001). This effect was even greater when combined with loop diuretics, which reduced blood volume after 14 days (p=0.035). Empagliflozin did not cause potassium wasting (p=0.20) or renal dysfunction (p>0.11 for all biomarkers). Empagliflozin may offer a beneficial diuretic effect for HF patients, potentially contributing to its positive long-term outcomes in this population.
Butt et al., 2022 [17]	Efficacy of Dapagliflozin in reducing HF progression, complications and death according to modified HF collaboratory score	RCT (DAPA-HF)	4,744	Dapagliflozin 10 mg once a day vs placebo. Follow-up visits were scheduled at 14, 60, and 120 days and then every 4 months thereafter	Compared to placebo, Dapagliflozin reduces the risk of cardiac death and deterioration of heart failure. mHFC score HRs from lowest to highest tertile were: 0.76 (95% confidence interval (CI) : 0.61-0.94), 0.76 (95% CI: 0.60-0.97), and 0.71 (95% CI: 0.55-0.90). No relation could be found between treatment efficacy and mHFC score (P= 0.89). Dapagliflozin has a consistent effect on reducing New York Heart Association (functional classification system for heart failure) (NYHA) class across different modified heart failure collaboration score (mHFC) tertiles (p=0.89).
Pitt et al., 2023 [18]	To investigate the efficacy of Sotagliflozin when started after HF hospitalization due to decompensation	RCT SOLOIST-WHF trial	1,222	Sotagliflozin 200 mg daily vs placebo. Median of 9 months	The primary endpoint which was defined to be cardiac death events found to be lower in the Sotagliflozin group compared to the placebo. Outcomes are significant, CI 0.52 to 0.85; p<0.001.
Berg et al., 2019 [19]	Creation of clinical risk assessment tool for HF hospitalization and evaluation for its ability to identify high-risk groups and efficacy of SGLT2i	Multicenter study of RCTs	8,578 from DECLARE-TIMI 58 and 8,212 from SAVOR-TIMI 53	Dapagliflozin was administered at 10 mg daily vs placebo. Median follow-up of 4.2 years.	Dapagliflozin reduced the risk of HHF in patients with T2DM. The absolute risk reduction was greater in patients with a higher baseline risk of HHF. A novel risk score was developed to identify patients with T2DM at high risk for hospitalization for heart failure (HHF). This score can be used to identify patients who may benefit most from treatment with SGLT2 inhibitors like Dapagliflozin.
Kato et al., 2019 [20]	To investigate the impact of Dapagliflozin on worsening left ventricular ejection fraction in diabetic patients with HF with and without reduced EF.	RCT DECLARE-TIMI 58	17,160	Dapagliflozin 10 mg daily or placebo. Median of 4.2 years of follow-up	Dapagliflozin generally reduces hospitalization due to HF and cardiac death (95% CI, 0.73-0.95; p=0.005); however, it was found to be more effective in reducing these risks in HF patients with reduced ejection fraction (EF) compared to patients with preserved EF (p=0.046).
Solomon et al., 2022 [21]	To compare Dapagliflozin's cardioprotective effect in diabetic patients with preserved EF with the well-established protective effect of it in patients with EF <60%	RCT	6,263	Dapagliflozin 10 mg daily or matching placebo. Median 2.3 years	Dapagliflozin showed a reducing effect on the primary endpoint; worsening EF and cardiac death, in the overall population which when compared to patients with EF <60, was found similar with no changes related to EF initial value (p=0.009).
Furtado et al., 2019 [22]	To investigate the effect of Dapagliflozin in diabetic patients with and without previous MI events on future cardiac outcomes regarding hospitalizations and cardiac deaths.	Subanalysis from the DECLARE-TIMI 58 Trial	17,160	Dapagliflozin 10 mg daily vs placebo. Median of 4.2 years of follow-up	Dapagliflozin was highly effective as cardio-protective in patients with previous MI and MACE (95% CI, 0.72–0.99; p=0.039), however, not significantly protective in patients without previous CV events (95% CI, 0.88–1.13; p=0.97).
Wiviott et al., 2019 [23]	To evaluate the safety and efficacy of Dapagliflozin compared to placebo in patients with T2DM	DECLARE–TIMI 58 trial RCT	17,160 patients, including 10,186 without atherosclerotic CV disease	Dapagliflozin 10 mg daily vs placebo. Median follow-up 4.2 years	Dapagliflozin was as safe as placebo in terms of major adverse CV events (MACE) (p-value for non-inferiority<0.001). No significant reduction in overall MACE compared to placebo (p=0.17). Significantly reduced rate of CV death or hospitalization for HF compared to placebo (p=0.005), driven by lower hospitalization rates for heart failure. Some potential benefits for kidney function compared to placebo (p=0.76). No significant impact on the overall death rate compared to the placebo (p=0.93). Increased risk of diabetic ketoacidosis compared to placebo (p=0.02). Higher rate of genital infections compared to placebo (p<0.001). Dapagliflozin did not increase the risk of MACE compared to placebo but did offer some CV benefits by reducing the risk of hospitalization for HF.
Anker et al., 2021 [24]	The effects of empagliflozin on HF and kidney function in patients with chronic HF and a reduced ejection fraction (HFrEF)	A secondary analysis of EMPEROR-Reduced trial RCT	3,730 patients with HFrEF. Roughly half (50%) had diabetes, 34% had prediabetes, and 16% were normoglycemic	Empagliflozin (10 mg daily) vs placebo in addition to their usual HF medications. Median follow-up 20 months	Empagliflozin reduced CV death/hospitalization (p<0.001) for HF compared to placebo, regardless of diabetes status. Fewer HF hospitalizations (p<0.05) in all patient groups. Slower kidney decline (p<0.05) across all groups, with a possible greater benefit in diabetic patients. Improved kidney outcomes (p<0.05) in all groups, regardless of diabetes status. Empagliflozin only lowered blood sugar in diabetic patients and did not increase the risk of low blood sugar in any group.
Lee et al., 2021 [25]	To assess the effects of empagliflozin, on cardiac structure and function in patients with HF with reduced (HFrEF) and T2DM or prediabetes.	(SUGAR-DM-HF) RCT	105	Empagliflozin 10 mg once daily or a matching placebo. Follow-up for 36 weeks	(LVESVi): Decreased by 7.9 mL/m^2^ in the empagliflozin group vs 1.5 mL/m^2^ in the placebo group (p=0.015) (LV GLS): No significant difference between groups; secondary outcomes showed a decrease in LVEDVi with empagliflozin compared to placebo (p=0.004), but no other significant changes in other cardiac MRI parameters, clinical outcomes, or biomarkers.
Kramer et al., 2023 [26]	Evaluate changes in hemodynamic markers as mediators of CV and kidney benefits with empagliflozin	Post hoc analysis of EMPA-REG OUTCOME trial RCT	7,020	Empagliflozin (10 and 25 mg) vs placebo in addition to standard of care. Follow-up median of 3.1 years	Empagliflozin treatment at week 12 improved markers of arterial stiffness, vascular resistance, and cardiac workload in patients with T2DM and established CV disease, but effects on these variables did not appear to largely mediate the benefits of empagliflozin in CV, HF, and kidney outcomes.
Sen et al., 2021 [27]	The effect of canagliflozin on circulating GDF‐15, CV events, hospitalization for HF, and kidney outcomes in patients with T2DM	Post hoc analysis of the CANVAS trial RCT	4,330	100 mg canagliflozin, 300 mg canagliflozin, vs placebo. Median follow‐up of 6.1 years	Higher GDF-15 levels linked to increased risk of CV outcomes (HR=1.2, 95% CI: 1.0-1.3). HF (HR=1.5, 95% CI: 1.2-2.0). Kidney problems (HR=1.5, 95% CI: 1.2-2.0). Canagliflozin modestly lowered GDF-15, but this did not explain the drug's benefit on CV and kidney health.
Ferreira et al., 2021 [28]	Evaluation of cardio/kidney composite endpoints by 2 statistical approaches in T2DM patients treated with Empagliflozin	Post hoc analysis of EMPA-REG OUTCOME trial RCT	7,020	Empagliflozin (10 and 25 mg) vs placebo in addition to standard of care. Follow-up median of 3.1 years	Empagliflozin significantly reduced the risk of combined CV and kidney complications (hazard ratio (HR): 0.56, 95% CI: 0.49-0.64).
Bhatt et al., 2021 [29]	The efficacy and safety of Sotagliflozin on patients with T2DM who were recently hospitalized for worsening HF	(SOLOIST-WHF) trial RCT	1,222	Sotagliflozin 200 mg (with a possible dose increase to 400mg) vs placebo. Follow-up for a median of 9.0 months	Sotagliflozin therapy, initiated before or shortly after discharge, resulted in a significantly lower total number of deaths from CV causes and hospitalizations and urgent visits for HF than placebo. The rate of the primary endpoint was 51.0 in the Sotagliflozin group compared to 76.3 in the placebo group (p<0.001).
Nassif et al., 2019 [30]	Effects of Dapagliflozin on biomarkers, symptoms, and functional status in patients with HF with reduced EF inpatients with and without T2DM	DEFINE-HF trial RCT	263	Dapagliflozin 10 mg daily vs placebo 12 weeks	Average levels of NT-proBNP did not differ significantly between dapagliflozin and placebo groups after 6 or 12 weeks (p=0.43). Dapagliflozin group improvement in health status: Measured by Kansas City Cardiomyopathy Questionnaire (KCCQ) score (≥5-point increase) (p=0.039) OR ≥20% reduction in NT-proBNP levels. Positive effects of dapagliflozin were observed in patients with and without T2DM.
Norhammar et al., 2019 [4]	To investigate CV safety and event rates for dapagliflozin versus other glucose-lowering drugs (GLDs) in a real-world T2DM population	A nationwide observational study Retrospective Cohort	28,408	Dapagliflozin 10 mg vs other glucose-lowering drugs (GLDs). Patients were observed until death or the end of the study period (2013-December 31, 2016)	Dapagliflozin was associated with a 21% lower risk of HHF or CV mortality versus other GLDs, with no significant association with MACE and CV mortality risks, MI, and stroke.
Real et al., 2021 [31]	To evaluate CV and mortality benefits of SGLT2i in patients with T2DM	CVD-REAL Catalonia a retrospective cohort study	12,917	Any SGLT-2i (i.e., canagliflozin, dapagliflozin, or empagliflozin) or oGLDs. Data between January 2013 and December 2016	The use of SGLT2i was associated with a lower risk of heart failure (p<0.001), all-cause death (p<0.001), all-cause death or heart failure (p<0.001), modified MACE (p<0.001), chronic kidney disease (p<0.001).
Jariwala et al., 2021 [32]	To investigate impact of the addition of SGLT2-i to ARNI therapy in patient with refractory heart failure regardless of their diabetic status	Retrospective cohort study	104	Dapagliflozin, Data between January 2020 and June 2020	Significant change in left ventricular function, mean change +9.00 ± 0.62 was noticed to associate the addition of Dapagliflozin to ARNI therapy after 6 months of starting it (p<0.001). Dual therapy of ARNI and Dapagliflozin resulted in significant improvement of median NYHA classification by 2.3 (95% confidence interval: 2.245-2.355).

Discussion

This systematic review sheds light on the potential of SGLT-2 inhibitors to influence cardiovascular health in adult patients with T2DM. We leverage a comprehensive body of evidence, incorporating data from 22 RCTs and three observational cohort studies. This combined approach offers a robust foundation for evaluating the cardiovascular impact of SGLT-2 inhibitors. The RCT component of our analysis focuses on the cardiovascular effects of various SGLT-2 inhibitors (canagliflozin, dapagliflozin, empagliflozin, ertugliflozin, sotagliflozin) in T2DM patients. These 22 trials employed treatment durations ranging from three months to four years.

The RCTs investigated various outcomes, including changes in heart failure biomarkers, exercise hemodynamics, functional capacity, left ventricular remodeling, and major adverse cardiovascular events (MACE). Several studies further explored these effects in specific subgroups (e.g., different durations of HF or varying degrees of ejection fraction) through post hoc analyses.

Several studies demonstrated favorable effects of SGLT-2 inhibitors on cardiovascular outcomes. Empagliflozin significantly reduced the risk of cardiovascular death, HF hospitalization, and MACE in patients with T2DM, regardless of the control of other cardiovascular risk factors at baseline [12,28]. Dapagliflozin also showed consistent benefits across different subgroups [13,19,20,21]. Similarly, sotagliflozin reduced the risk of cardiac death and HF events [18,29]. The DAPA-HF further demonstrated consistent benefits of dapagliflozin on heart failure outcomes across different risk profiles [17].

The mechanisms underlying the cardiovascular benefits of SGLT-2 inhibitors are multifaceted. Several studies demonstrated improvements in hemodynamic parameters, such as reduced left ventricular filling pressure, blood pressure, and right ventricular systolic pressure during exercise [10,16]. Additionally, SGLT-2 inhibitors were found to have diuretic properties, which may contribute to their positive effects on HF outcomes [16]. However, the extent to which these hemodynamic changes influence long-term cardiovascular and renal benefits remains under investigation [26].

It is important to note that not all studies showed consistent positive results. Some trials, like CANDLE [9] and REFORM [14], did not demonstrate conclusive benefits for all investigated outcomes. These findings highlight the importance of further research. Furthermore, some studies highlighted the importance of patient characteristics in determining the efficacy of SGLT-2 inhibitors. For example, the DECLARE-TIMI 58 trial found that dapagliflozin was more effective in reducing cardiovascular events in patients with a history of myocardial infarction [22,23].

The EMPEROR-Reduced trial demonstrated that empagliflozin reduced cardiovascular death or hospitalization for heart failure in patients with chronic HF and reduced ejection fraction, regardless of their diabetes status [24]. These findings suggest potential benefits beyond glycemic control.

The VERTIS CV trial examined the effects of ertugliflozin and found no increased risk of cardiovascular or kidney problems compared to the placebo. However, it showed reduced HF hospitalizations and a slower decline in kidney function [15]. This suggests that the cardiovascular and renal benefits of SGLT-2 inhibitors vary between individual drugs. Moreover, the CANVAS trial [27] found that higher baseline levels of growth differentiation factor-15 (GDF-15), a biomarker associated with cardiovascular and kidney risk, were linked to an increased risk of these outcomes. While canagliflozin modestly lowered GDF-15 levels, this did not fully explain the drug's cardiovascular and kidney benefits.

Several studies explored the impact of SGLT-2 inhibitors on HF-specific outcomes. The DAPA-VO2 trial demonstrated that dapagliflozin significantly improved exercise capacity in patients with HF and reduced ejection fraction [11]. The SUGAR-DM-HF trial found that empagliflozin improved left ventricular remodeling in patients with HF and T2DM or prediabetes [25]. The DEFINE-HF trial showed that dapagliflozin improved health status in patients with HF, suggesting benefits beyond blood sugar control [30]. These findings highlight the potential of SGLT-2 inhibitors for improving heart failure outcomes.

The 22 RCT studies included in this review provide a comprehensive understanding of the cardiovascular and HF-specific benefits of SGLT-2 inhibitors in patients with T2DM. While not all studies showed consistent positive results, most of the evidence supports using these medications for improving cardiovascular and HF outcomes, even in high-risk patient populations. The mechanisms appear multifaceted, involving both hemodynamic and metabolic effects, and may vary between individual SGLT-2 inhibitors.

The three included observational studies in this review, which are retrospective cohort studies, also showed positive cardiovascular benefits of SGLT-2 inhibitors compared to other hypoglycemic agents in patients with T2DM. A nationwide study showed that Dapagliflozin has a 21% lower risk of HHF or CV mortality than other hypoglycemic agents. It is also proved that Dapagliflozin does not increase the risk of MACE, myocardial infarction (MI), or other CV morbidities and mortality [4].

Moreover, the CVD-REAL had a positive outcome of lowering the risk of heart failure progression and death in patients with T2DM who were prescribed SGLT-2 inhibitors, which were statistically significant [31]. Adding SGLT-2 inhibitors to angiotensin receptor blocker-neprilysin inhibitors (ARNI) also showed a significant lowering of NYHA classification in patients with refractory heart failure besides improvement of left ventricular function with mean change +9.00 ± 0.62 [32].

By analyzing data from 22 RCTs and three observational cohort studies, we established a robust foundation for understanding the cardiovascular benefits of these medications. Our systematic review highlights the promise of SGLT-2 inhibitors for improving cardiovascular health in T2DM patients. While some variations exist, the evidence suggests these medications reduce cardiovascular death, hospitalization for heart failure, and major adverse cardiovascular events. Future research should focus on refining treatment strategies and understanding the long-term effects of SGLT-2 inhibitors.

Strengths of the Review

This systematic review offers a comprehensive analysis of the impact of SGLT-2 inhibitors on heart failure in patients with type 2 diabetes mellitus. The review's strengths are its meticulous methodology, rigorous quality assessment, and transparent reporting. Firstly, the review employed a comprehensive search strategy. It meticulously searched across multiple major databases and clinical trial registries, ensuring a broad capture of relevant studies.

The search strategy utilized a combination of MeSH terms and keywords, further enhancing its ability to encompass a wide range of literature on the topic. Additionally, a detailed search strategy is provided, fostering transparency and allowing for replication of the review process by others.

Secondly, the review established rigorous inclusion and exclusion criteria. These pre-defined criteria serve as a safeguard, ensuring the eligibility and relevance of the included studies. The criteria focused explicitly on the target population (patients with type 2 diabetes and heart failure), the intervention of interest (SGLT-2 inhibitors), and the type of publications considered (original research). This focused approach helps to minimize bias and guarantees that the review findings are directly applicable to the research question.

Furthermore, the review demonstrates a commitment to thorough quality assessment. It implemented appropriate tools to meticulously assess the methodological quality and potential for bias within the included studies. For RCTs, the gold standard for evaluating interventions, the review prioritized the inclusion of 22 such studies. This focus on high-quality evidence strengthens the overall confidence in the review's findings. Moreover, the review employed appropriate tools like the Cochrane Risk of Bias 2 tool for RCTs and the Newcastle-Ottawa Scale for observational studies. Studies with a high risk of bias were excluded, further ensuring the reliability of the synthesized evidence.

The review acknowledges the anticipated heterogeneity in the study designs, populations, and interventions. To address this, it outlines a plan for a narrative synthesis. This approach allows for a comprehensive and qualitative summary of the available evidence, providing a richer understanding than solely relying on quantitative meta-analysis.

Finally, the review prioritizes transparency and reproducibility. The adherence to the PRISMA 2020 guidelines significantly enhances the transparency and reporting quality of the systematic review. The detailed search strategy, inclusion/exclusion criteria, and quality assessment methods are all clearly documented within the review. Furthermore, the use of standardized data extraction forms and reference management software further contributes to the overall transparency and rigor of the review process.

Limitations of the Review and Included Studies

This systematic review provides valuable information on SGLT-2 inhibitors for heart failure in T2DM. However, acknowledging potential limitations strengthens the review's transparency and allows for a more nuanced interpretation of the findings.

One limitation lies in the review's scope. While focusing on SGLT-2 inhibitors and heart failure is informative, including other diabetes medications or cardiovascular outcomes could offer a broader understanding. Additionally, the review might be affected by heterogeneity in the included studies' designs, populations, and outcome measures. This heterogeneity can make synthesizing evidence and drawing definitive conclusions challenging, potentially limiting the generalizability of the findings. Furthermore, restricting the search to English-language publications might introduce language bias, potentially excluding relevant research and creating gaps in the evidence synthesis.

Limitations inherent to the included studies themselves also warrant consideration. The studies may encompass a mix of designs, ranging from randomized controlled trials (considered the gold standard) to observational studies, each with strengths and weaknesses. This heterogeneity can complicate comparisons and syntheses of the findings. Selection bias within the included studies is another potential limitation. Recruitment of participants with specific characteristics or exclusion of certain subgroups could limit the generalizability of the findings to the broader T2DM and heart failure population.

Incomplete reporting of relevant outcomes, such as heart failure-related hospitalizations, mortality, or quality of life, within some studies could hinder a comprehensive understanding of the full impact of SGLT-2 inhibitors. Additionally, short follow-up periods in the included studies might be insufficient to capture the long-term effects of these medications on heart failure progression and other clinically significant outcomes. Ideally, future studies would have extended follow-up periods.

Finally, the review's lack of direct comparisons between different SGLT-2 inhibitors limits the ability to determine their relative efficacy and safety. Comparative studies would provide valuable insights into the differential impacts of individual SGLT-2 inhibitors on heart failure outcomes. By acknowledging and discussing these limitations, the review fosters a more critical appraisal of the findings and identifies areas where further research is necessary. This transparency strengthens the overall value of the systematic review.

Future Directions for Research on SGLT-2 Inhibitors and Heart Failure in T2DM

Limitations identified in the systematic review highlight critical areas for future research on SGLT-2 inhibitors and heart failure in T2DM. Broader outcome assessments, encompassing cardiovascular mortality, quality of life, and a more comprehensive range of events, are crucial to understanding the overall benefits. Comparative studies directly comparing different SGLT-2 inhibitors would inform optimal treatment selection.

Extending follow-up periods in future studies is essential to assess the sustained impact of SGLT-2 inhibitors on heart failure progression and mortality. Investigating the differential effects of these medications in specific patient subgroups, such as those with varying ejection fraction or risk profiles, can identify who benefits the most.

Real-world studies complementing randomized trials are valuable to bridge the gap between research and clinical practice. Additionally, mechanistic studies exploring the cardioprotective mechanisms of SGLT-2 inhibitors can enhance our understanding of their therapeutic potential. Finally, including more diverse patient populations in future research ensures the generalizability of findings and identification of potential disparities in treatment responses. By addressing these future research directions, we can solidify the evidence base and optimize clinical decision-making for managing heart failure in T2DM patients using SGLT-2 inhibitors.

## Conclusions

This comprehensive systematic review provides valuable insights into the impact of SGLT-2 inhibitors on heart failure outcomes in patients with T2DM. The rigorous search strategy and quality assessment of the included studies strengthen the credibility of the findings. The review synthesizes evidence suggesting that SGLT-2 inhibitors offer a beneficial effect in reducing heart failure-related hospitalizations and potentially improving other cardiovascular outcomes, including mortality and quality of life. These findings are particularly significant given the high prevalence of heart failure in the diabetic population and the substantial burden associated with this comorbidity.

While acknowledging the limitations of the existing research, the review highlights promising avenues for future exploration. More extensive, long-term studies employing head-to-head comparisons between different SGLT-2 inhibitors would be valuable. Additionally, investigating the effects of SGLT-2 inhibitors in diverse patient subgroups can provide a more comprehensive understanding of their clinical utility in managing heart failure within the T2DM population. By pursuing these future research directions, we can further refine our knowledge and translate it into optimal clinical decision-making for patients with T2DM and heart failure.
